# Low Polymerase Activity Attributed to PA Drives the Acquisition of the PB2 E627K Mutation of H7N9 Avian Influenza Virus in Mammals

**DOI:** 10.1128/mBio.01162-19

**Published:** 2019-06-18

**Authors:** Libin Liang, Li Jiang, Junping Li, Qingqing Zhao, Jinguang Wang, Xijun He, Shanyu Huang, Qian Wang, Yuhui Zhao, Guangwen Wang, Nan Sun, Guohua Deng, Jianzhong Shi, Guobin Tian, Xianying Zeng, Yongping Jiang, Liling Liu, Jinxiong Liu, Pucheng Chen, Zhigao Bu, Yoshihiro Kawaoka, Hualan Chen, Chengjun Li

**Affiliations:** a State Key Laboratory of Veterinary Biotechnology, Harbin Veterinary Research Institute, Chinese Academy of Agricultural Sciences, Harbin, People’s Republic of China; b Division of Virology, Department of Microbiology and Immunology, Institute of Medical Science, University of Tokyo, Tokyo, Japan; University of Pittsburgh School of Medicine; Exotic & Emerging Avian Viral Diseases Research Unit; The Peter Doherty Institute for Infection and Immunity

**Keywords:** ANP32A, avian influenza virus, H7N9, PB2 E627K mutation, viral PA protein, viral adaptation in mammals, viral polymerase

## Abstract

Avian influenza viruses (AIVs) must acquire mammalian-adaptive mutations before they can efficiently replicate in and transmit among humans. The PB2 E627K mutation is known to play a prominent role in the mammalian adaptation of AIVs. The H7N9 AIVs that emerged in 2013 in China easily acquired the PB2 E627K mutation upon replication in humans. Here, we generate a series of reassortant or mutant H7N9 AIVs and test them in mice. We show that the low polymerase activity attributed to the viral PA protein is the intrinsic driving force behind the emergence of PB2 E627K during H7N9 AIV replication in mice. Four residues in the N-terminal region of PA are critical in mediating the PB2 E627K acquisition. Notably, due to the identity of viral PA protein, the polymerase activity and growth of H7N9 AIV are highly sensitive to changes in expression levels of human ANP32A protein. Furthermore, the impaired viral polymerase activity of H7N9 AIV caused by the depletion of ANP32A led to reduced virus replication in *Anp32a^−/−^* mice, abolishing the acquisition of the PB2 E627K mutation and instead driving the virus to acquire the alternative PB2 D701N mutation. Taken together, our findings show that the emergence of the PB2 E627K mutation of H7N9 AIV is driven by the intrinsic low polymerase activity conferred by the viral PA protein, which also involves the engagement of mammalian ANP32A.

## Abstract (precis)

The emergence of the PB2 E627K substitution is critical in the mammalian adaptation and pathogenesis of AIV. H7N9 AIVs that emerged in 2013 possess a prominent ability in gaining the PB2 E627K mutation in humans. Here, we demonstrate that the acquisition of the H7N9 PB2 E627K mutation is driven by the low polymerase activity conferred by the viral PA protein in human cells, and four PA residues are collectively involved in this process. Notably, the H7N9 PA protein leads to significant dependence of viral polymerase function on human ANP32A protein, and *Anp32a* knockout abolishes PB2 E627K acquisition in mice. These findings reveal that viral PA and host ANP32A are crucial for the emergence of PB2 E627K during adaptation of H7N9 AIVs to humans.

## INTRODUCTION

Avian species are the natural hosts of influenza A viruses, which continuously challenge the poultry industry and human health. Avian influenza viruses (AIVs) must obtain a series of mutations in their genomes to become adapted to mammalian hosts before they can efficiently replicate in and transmit among humans (1). Mutations in the hemagglutinin (HA) and basic polymerase 2 (PB2) proteins are particularly important. Amino acid mutations in HA are known to play important roles in promoting efficient binding to human-type receptors, thereby facilitating the replication and transmission of influenza virus in humans (2–5). Using a ferret model, Lakdawala et al. showed that transmissible influenza viruses with human-type receptor preference are rapidly selected in the soft palate, where long-chain α2,6-sialic acids predominate on the nasopharyngeal surface (6). Different mutations in PB2 have been identified to contribute to the adaptation of influenza viruses to mammalian hosts; these mutations include E627K (7, 8), D701N (9, 10), T271A (11), Q591R/K (12, 13), and E158G (14).

The key role of the PB2 E627K substitution in the mammalian adaptation of AIVs has been well documented, having been shown to enhance polymerase activity, virus replication, pathogenicity, and transmission of AIVs in humans and other mammals (2, 7, 8, 15–19). However, not all AIVs acquire the PB2 E627K mutation upon infection of humans, such as many H5N1 viruses (20, 21). Therefore, understanding the biological mechanism involved in the occurrence of this substitution is important to gain insights into the adaptation and pathogenesis of AIVs in mammals.

In early 2013 in China, a novel H7N9 AIV crossed the species barrier and caused the first human infection (22). To date, five epidemic waves of human infection with H7N9 viruses have caused 1,567 cases, with a fatality rate of approximately 39% (23). Moreover, highly pathogenic H7N9 viruses possessing a multibasic cleavage site motif in HA were detected in 2017 and caused severe threats to the poultry industry and human health (18, 24–26). Remarkably, the PB2 of H7N9 viruses easily acquires the PB2 E627K or D701N adaptive mutation during replication in mammalian hosts (18, 22, 27). In particular, most H7N9 human isolates acquire the PB2 E627K substitution (18). The prominent ability of H7N9 AIVs to acquire the PB2 E627K substitution upon infection of humans provides us with an excellent opportunity to decipher the underlying mechanism by which AIVs rapidly acquire the PB2 E627K substitution during their adaptation to mammalian hosts, including humans.

H7N9 AIV is a triple reassortant virus, with all six internal genes being derived from H9N2 AIVs (19, 28). In this study, we generated a series of reassortant or mutant viruses in the background of an avian 2013 H7N9 virus, harboring genes or mutations from an avian H9N2 virus, and monitored the residue phenotype of PB2 627 during viral passages in mice. We found that the low polymerase activity attributed to the H7N9 viral PA protein is the intrinsic force driving the emergence of the PB2 E627K mutation during the replication of H7N9 AIVs in mammals, a process that also involves interplay with host factors, such as ANP32A.

## RESULTS

### Different avian influenza viruses have different capabilities to acquire the PB2 E627K mutation during replication in MDCK cells and mice.

To investigate whether the H7N9 and H9N2 viruses have the same capability to obtain the PB2 E627K mutation during replication in a mammalian host, we first selected an H7N9 avian strain, A/pigeon/Shanghai/S1421/2013 [PG/S1421(H7N9)], and five H9N2 avian strains and passaged them in MDCK cells. As shown in Table 1, two H9N2 viruses, A/chicken/Henan/5/1998 [CK/5(H9N2)] and A/chicken/Guangxi/9/1999 [CK/9(H9N2)], isolated in the 1990s (29) did not acquire the PB2 E627K mutation even after being passaged six times in MDCK cells. Three other H9N2 viruses and the PG/S1421(H7N9) virus obtained the PB2 E627K mutation at passages 3 to 6 in MDCK cells (Table 1). The phenotypic emergence of PB2 E627K mutation was confirmed as occurring during virus passage because deep sequencing did not identify any subpopulations of PB2 627K in the original stocks of PG/S1421(H7N9) (see Table S1 in the supplemental material) and CK/SC197(H9N2) and CK/SC324(H9N2) virus (30).

**TABLE 1:** Different avian influenza viruses differ in their capability to acquire the PB2 E627K mutation during serial passages in MDCK cells and mice

Virus [abbreviation]	No. of passages for the virus to acquire PB2 E627K mutation	
	In MDCK cells^*a*^	In mice^*b*^^,^^*c*^
A/chicken/Henan/5/1998 [CK/5(H9N2)]	>6	>4 (0/3)
A/chicken/Guangxi/9/1999 [CK/9(H9N2)]	>6	2 (3/3)
A/chicken/Guangxi/C2163/2012 [CK/C2163(H9N2)]	3	ND^*d*^
A/chicken/Shanghai/SC197/2013 [CK/SC197(H9N2)]	6	ND
A/chicken/Zhejiang/SC324/2013 [CK/SC324(H9N2)]	4	ND
A/pigeon/Shanghai/S1421/2013 [PG/S1421(H7N9)]	4	1 (3/3)

a Viral RNAs were isolated 48 h postinfection (p.i.), and the PB2 segment was amplified by RT-PCR and sequenced to monitor the adaptive mutations arising during passages in MDCK cells.

b Three 6-week-old female BALB/c mice were intranasally inoculated with 10^6^ EID_50_ of each virus in a 50-μl volume. Viral RNAs were isolated from the lung homogenates of three infected mice on day 5 p.i., and the PB2 segment was amplified by RT-PCR and sequenced to monitor adaptive mutations arising during passages in mice.

c The number of mice harboring the PB2 E627K mutation in the indicated passage is shown in parentheses.

d ND, not done.

10.1128/mBio.01162-19.7 TABLE S1 Deep-sequencing analysis of key adaptive positions in the PB2 gene of viruses recovered from the lungs of mice that were inoculated with the indicated virus. ^a^Three 6-week-old female BALB/c mice from each group were inoculated with the indicated virus, and their lungs were collected on day 5 postinfection (p.i.). Viruses recovered from the lung homogenate mixture of the three mice were reinoculated into three more mice. Viral RNAs isolated from lung homogenates of the three second-passage mice on day 5 p.i. as well as viral RNAs isolated from the stock viruses were deep sequenced. ^b^Frequencies of <0.1% are denoted as 0. Download Table S1, PDF file, 0.05 MB. Copyright © 2019 Liang et al. 2019 Liang et al. This content is distributed under the terms of the Creative Commons Attribution 4.0 International license.

Next, we determined the residue phenotype of PB2 627 for PG/S1421(H7N9) and the two H9N2 strains, CK/5(H9N2) and CK/9(H9N2), during *in vivo* passages in mice. PG/S1421(H7N9) quickly acquired the PB2 E627K mutation on day 5 after one passage in mice, CK/9(H9N2) acquired the PB2 E627K mutation after the second passage, whereas for CK/5(H9N2), the PB2 627E residue was stably maintained even after four passages in mice (Table 1). CK/5(H9N2), a representative of viruses that maintain a stable PB2 627E during replication in mammals, was selected with PG/S1421(H7N9) to form a model virus pair to investigate the viral factors that drive the emergence of the PB2 E627K mutation during adaptation of H7N9 viruses to mammalian hosts.

### The low polymerase activity attributed to PA drives the emergence of the mammalian-adaptive PB2 E627K mutation.

**FIG 1:**
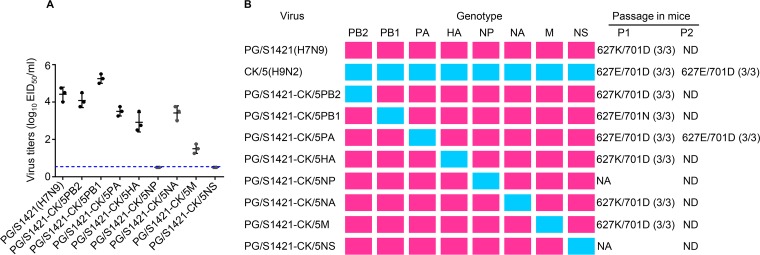
PA drives the emergence of the PB2 E627K mutation during the replication of PG/S1421(H7N9) virus in mice. (A) Viral titers in the lungs of mice (*n* = 3) on day 5 postinfection (p.i.) with 10^6^ EID_50_ of the tested viruses. The dashed line indicates the lower limit of detection. (B) Mammalian-adaptive mutations in the PB2 protein of the reassortant viruses acquired during passages in mice. Viral RNAs were isolated from lung homogenates of infected mice (*n* = 3) on day 5 p.i., and the PB2 segment was amplified by RT-PCR and sequenced to monitor adaptive mutations arising during passages in mice. The number of mice harboring the indicated PB2 residue is shown in parentheses. NA, virus was not recovered; ND, not done.

We used PG/S1421(H7N9) as the backbone to generate reassortant viruses, each containing one gene derived from CK/5(H9N2), as described previously (31). The rescued single-gene reassortant viruses were intranasally inoculated into mice to monitor their ability to acquire the PB2 E627K mutation. As shown in Fig. 1A, the reassortants exhibited variable replicative abilities in the lungs of infected mice: the reassortant containing CK/5(H9N2) PB1 replicated to higher titers than parental PG/S1421(H7N9); the replication titers of reassortants containing the PB2, PA, HA, NA, or M protein of CK/5(H9N2) were lower than that of PG/S1421(H7N9); and the reassortants containing the NP or NS of CK/5(H9N2) replicated poorly in the lungs of infected mice with no virus detected on day 5 postinfection (p.i.). We then sequenced the PB2 gene of the reassortant viruses recovered from the lungs of the infected mice. The reassortants containing the PB2, HA, NA, or M protein of CK/5(H9N2) acquired the PB2 E627K mutation on day 5 p.i. in mice, just like parental PG/S1421(H7N9) (Fig. 1B). The reassortant virus containing CK/5(H9N2) PB1 obtained a PB2 D701N mutation on day 5 p.i. (Fig. 1B). In contrast, the reassortant virus bearing CK/5(H9N2) PA was stable during replication in mice with no mutations acquired in PB2 (Fig. 1B). The recovered PG/S1421-CK/5PA(H7N9) virus was then reinoculated into mice to identify potential adaptive mutations in PB2. Again, no mutations occurred in PB2 of PG/S1421-CK/5PA(H7N9) recovered from the second-passage mice (Fig. 1B). Deep sequencing of PG/S1421-CK/5PA(H7N9) recovered from the lungs of the second-passage mice demonstrated that the PB2 E627K mutation was not acquired (Table S1). These results indicate that the PA gene carried by PG/S1421(H7N9) is responsible for driving the emergence of the PB2 E627K mutation in mice and that replacing PG/S1421(H7N9) PA with CK/5(H9N2) PA abolishes the acquisition of the adaptive PB2 E627K mutation in the mammalian host.

The PA protein is a component of the viral ribonucleoprotein (RNP) complex, which is important for virus replication and virulence (32–36). We therefore performed a minigenome assay to determine the viral polymerase activity of four RNP combinations possessed by PG/S1421(H7N9), CK/5(H9N2), and two single PA reassortants in avian DF-1 cells. As shown in Fig. S1, the polymerase activity of PG/S1421(H7N9) (i.e., 7_PB2_7_PB1_7_PA_7_NP_) was much higher than that of CK/5(H9N2) (i.e., 9_PB2_9_PB1_9_PA_9_NP_) (the gene segments derived from the parental H7N9 virus were designated “7,” and those derived from the parental H9N2 virus were designated “9”), which may partially explain the prevalence of H7N9 viruses in avian species. Notably, the polymerase activity of 7_PB2_7_PB1_9_PA_7_NP_ was dramatically decreased compared with that of 7_PB2_7_PB1_7_PA_7_NP_, whereas the polymerase activity of 9_PB2_9_PB1_7_PA_9_NP_ was slightly increased compared with that of 9_PB2_9_PB1_9_PA_9_NP_. These data suggest that the PG/S1421(H7N9) PA is better able to confer high polymerase activity in an avian host, especially in the H7N9 virus background.

10.1128/mBio.01162-19.1 FIG S1 Polymerase activity of four RNP combinations possessed by PG/S1421(H7N9), CK/5(H9N2), and two single PA reassortants in avian DF-1 cells. Values shown are means ± SDs from triplicate transfections of a representative experiment and are standardized to the polymerase activity of PG/S1421(H7N9) (i.e., 7_PB2_7_PB1_7_PA_7_NP_). Viral gene segments derived from PG/S1421(H7N9) and CK/5(H9N2) are denoted as “7” and “9,” respectively. *P* values were determined by using a two-tailed unpaired Student's *t* test compared to the polymerase activity of 7_PB2_7_PB1_7_PA_7_NP_. Data are representative of three independent experiments. Download FIG S1, TIF file, 0.4 MB. Copyright © 2019 Liang et al. 2019 Liang et al. This content is distributed under the terms of the Creative Commons Attribution 4.0 International license.

Because our study in mice showed that PA is important for PB2 E627K acquisition during mammalian adaptation of PG/S1421(H7N9), we next investigated whether PG/S1421(H7N9), CK/5(H9N2), and their reassortants had different RNP activities in HEK293T cells (37). The polymerase activities varied markedly among the six RNP combinations possessed by PG/S1421(H7N9), CK/5(H9N2), and the eight single-gene reassortant viruses (Fig. S2A). For example, the polymerase activity of the RNP combination containing CK/5(H9N2) PA in the PG/S1421(H7N9) background (i.e., 7_PB2_7_PB1_9_PA_7_NP_) was approximately 12-fold higher than that of parental PG/S1421(H7N9) (i.e., 7_PB2_7_PB1_7_PA_7_NP_), which is the opposite of the observation in avian DF-1 cells. In contrast, the polymerase activities of RNP combinations 7_PB2_7_PB1_7_PA_9_NP_ and 9_PB2_7_PB1_7_PA_7_NP_ were significantly lower than that of 7_PB2_7_PB1_7_PA_7_NP._ We found similar diversity among the polymerase activities of the other 10 RNP combinations between PG/S1421(H7N9) and CK/5(H9N2) (Fig. S2B). Of note, PG/S1421(H7N9) or single-gene reassortant viruses with low polymerase activities (RNP combinations 7_PB2_7_PB1_7_PA_7_NP_, 7_PB2_7_PB1_7_PA_9_NP_, and 9_PB2_7_PB1_7_PA_7_NP_) either failed to replicate in mice (PG/S1421-CK/5NP and PG/S1421-CK/5NS) or acquired the PB2 E627K substitution (PG/S1421, PG/S1421-CK/5PB2, PG/S1421-CK/5HA, PG/S1421-CK/5NA, and PG/S1421-CK/5M) (Fig. 1B and Fig. S2C). These results suggest that the PA protein of PG/S1421(H7N9) is the causative viral factor for the low polymerase activity in human cells, which is associated with the emergence of the PB2 E627K substitution during virus replication in mice. Interestingly, the introduction of CK/5(H9N2) PB1 into the PG/S1421(H7N9) backbone yielded higher polymerase activity than that of parental PG/S1421(H7N9) and led to the appearance of another mammalian-adaptive marker, PB2 D701N (9, 10, 38–40), during the replication of PG/S1421-CK/5PB1(H7N9) in mice. Two additional reassortant viruses—PG/S1421-CK/5PB1+PA(H7N9), possessing high polymerase activity (i.e., 7_PB2_9_PB1_9_PA_7_NP_), and PG/S1421-CK/5PB2+PB1(H7N9), bearing low polymerase activity (i.e., 9_PB2_9_PB1_7_PA_7_NP_)—were generated (Fig. S2C). PG/S1421-CK/5PB1+PA(H7N9) maintained a stable PB2 627E, whereas PG/S1421-CK/5PB2+PB1(H7N9) acquired the PB2 E627K mutation during replication in mice (Table S2). These results confirm that the low polymerase activity in human cells attributed to PG/S1421(H7N9) PA is associated with the emergence of the mammalian-adaptive PB2 E627K mutation.

10.1128/mBio.01162-19.2 FIG S2 Polymerase activity of the 16 RNP combinations between PG/S1421(H7N9) and CK/5(H9N2) in HEK293T cells and the viruses tested in the mouse study. (A) Polymerase activities of the six RNP combinations possessed by PG/S1421(H7N9), CK/5(H9N2), and the eight reassortant viruses bearing a single gene from CK/5(H9N2) in the PG/S1421(H7N9) background. (B) Polymerase activities of the other 10 RNP combinations between PG/S1421(H7N9) and CK/5(H9N2). Values shown are means ± SDs from triplicate transfections of a representative experiment and are standardized to the polymerase activity of PG/S1421(H7N9) (i.e., 7_PB2_7_PB1_7_PA_7_NP_, indicated by the dashed line). *P* values were determined by using a two-tailed unpaired Student's *t* test compared to the RNP complex 7_PB2_7_PB1_7_PA_7_NP_ (A and B). (C)The parental and reassortant viruses tested in mice and their corresponding RNP combinations. Viral gene segments derived from PG/S1421(H7N9) and CK/5(H9N2) are denoted as “7” and “9,” respectively (A to C). Data are representative of three independent experiments (A and B). Download FIG S2, TIF file, 1.2 MB. Copyright © 2019 Liang et al. 2019 Liang et al. This content is distributed under the terms of the Creative Commons Attribution 4.0 International license.

10.1128/mBio.01162-19.8 TABLE S2 Phenotype of the PB2 627 residue in reassortant and mutant H7N9 viruses during passage in mice. ^a^Three 6-week-old female BALB/c mice from each group were inoculated with the indicated virus and then euthanized on day 5 postinfection. Viral RNAs were isolated from lung homogenates, and the PB2 segment was amplified by RT-PCR and sequenced to monitor the adaptive mutations arising during passage in mice. The number of mice harboring the indicated PB2 627 residue is shown in parentheses. Download Table S2, PDF file, 0.01 MB. Copyright © 2019 Liang et al. 2019 Liang et al. This content is distributed under the terms of the Creative Commons Attribution 4.0 International license.

### Four amino acids in the N-terminal PA domain are critical in mediating the acquisition of PB2 E627K.

**FIG 2:**
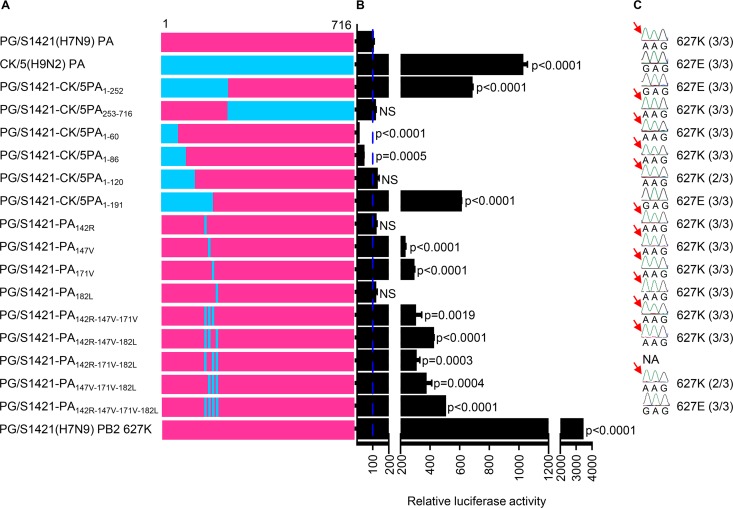
Four residues in the N-terminal PA domain mediate the acquisition of the H7N9 PB2 E627K mutation in mice. (A) Schematic diagram of PA chimeras between PG/S1421(H7N9) and CK/5(H9N2) as well as PA mutants in the background of PG/S1421(H7N9). (B) The effects of PA chimeras or mutants on viral polymerase activities in the background of PG/S1421(H7N9) in a minigenome assay. Four RNP expression plasmids (PG/S1421-PB2, -PB1, -NP, and wild-type, chimeric, or mutant PA) were transfected into HEK293T cells together with pHH21-SC09NS F-Luc and pRL-TK reporters. Thirty hours later, the cells were harvested for luciferase assays. The values are standardized to the polymerase activity of PG/S1421(H7N9) (100%). The polymerase activity of PG/S1421(H7N9) bearing the PB2 E627K mutation was also included as a control. *P* values were determined by using a two-tailed unpaired Student's *t* test compared to the RNP complex bearing PG/S1421(H7N9) PA. NS, not significant. (C) Sequencing of the PB2 627 domain during the replication of PG/S1421(H7N9) viruses bearing wild-type, chimeric, or mutant PAs in mice. Viral RNAs were isolated from lung homogenates of infected mice (*n* = 3) on day 5 p.i., and the PB2 segment was amplified by RT-PCR and sequenced to monitor adaptive mutations. The number of mice harboring the indicated PB2 residue is shown in parentheses. NA, virus was not recovered; red arrows indicate the appearance of the PB2 E627K mutation. Data are representative of three independent experiments (means ± SDs) (B).

To explore the specific domain of PG/S1421(H7N9) PA that mediates the emergence of the PB2 E627K mutation of H7N9 viruses, we created two PA chimeras in which domain 1–252 or domain 253–716 of PG/S1421(H7N9) PA was replaced with that of CK/5(H9N2) PA (Fig. 2A). The RNP combination containing domain 1–252 of CK/5(H9N2) PA (i.e., 7_PB2_7_PB1_7_9(1–252)PA_7_NP_) possessed significantly higher polymerase activity than that of 7_PB2_7_PB1_7_PA_7_NP_ in HEK293T cells, whereas the activity of the RNP combination containing domain 253–716 of CK/5(H9N2) PA (i.e., 7_PB2_7_PB1_7_9(253–716)PA_7_NP_) was as low as that of 7_PB2_7_PB1_7_PA_7_NP_ (Fig. 2B). Two chimeric PA viruses were then rescued in the background of PG/S1421(H7N9) and evaluated in mice. The chimeric PA virus containing domain 1–252 of CK/5(H9N2) PA replicated in mice without acquiring the PB2 E627K mutation (Fig. 2C). In contrast, the chimeric PA virus bearing domain 253–716 of CK/5(H9N2) PA acquired the PB2 E627K mutation during replication in mice (Fig. 2C). These results demonstrate that the N-terminal 1–252 domain of PA (PAN) is critical in mediating the acquisition of the PB2 E627K substitution during the replication of H7N9 virus in mammalian hosts.

**FIG 3:**
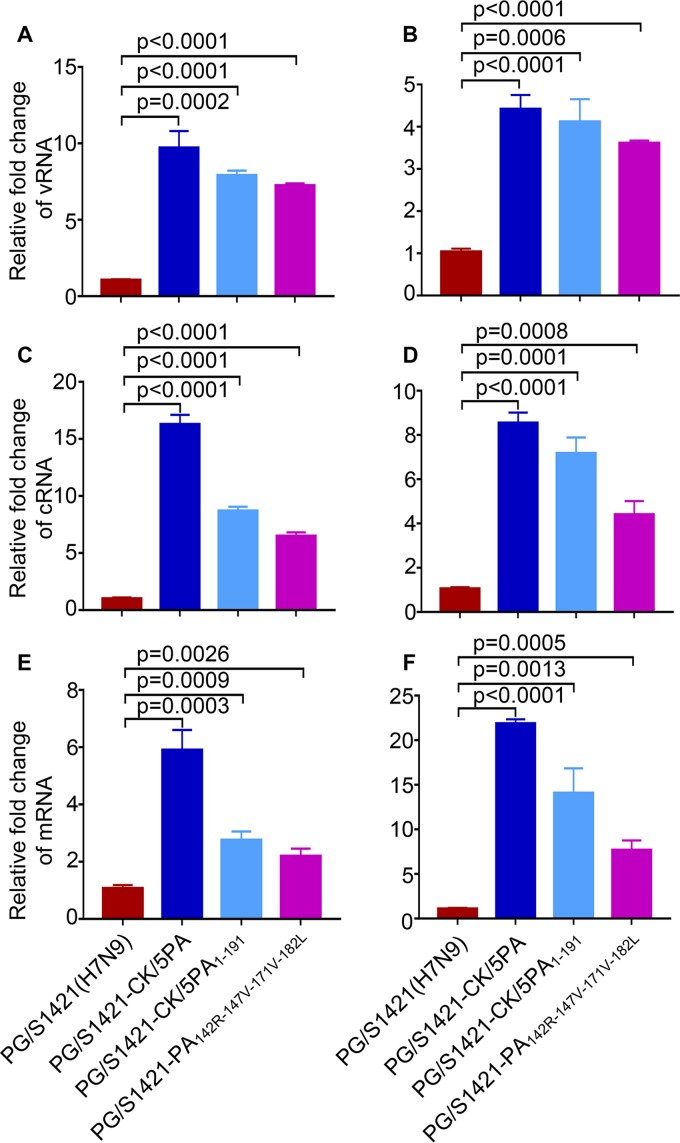
Replacement of H7N9 PA with chimeric or mutant PA enhances the transcription and replication of the viral genome in A549 cells. The levels of vRNA (A and B), cRNA (C and D), and mRNA (E and F) of the viral NP segment were determined by RT-qPCR in A549 cells that were infected for 6 h (A, C, and E) and 8 h (B, D, and F) with the PG/S1421(H7N9) viruses bearing different PAs. Values shown are means ± SDs from triplicates of a representative experiment, normalized to 18S RNA, and expressed as the fold change in comparison to the PG/S1421(H7N9) group. *P* values were determined by using a two-tailed unpaired Student's *t* test compared to levels of vRNA, cRNA, and mRNA in A549 cells infected with PG/S1421(H7N9) virus. Data are representative of three independent experiments (A to F).

Next, we constructed another four PA chimeras containing amino acids 1 to 60, 1 to 86, 1 to 120, or 1 to 191 of CK/5(H9N2) PA in the backbone of PG/S1421(H7N9) PA to identify the key region in PAN that determines the emergence of the PB2 E627K mutation (Fig. 2A). The polymerase activities of the three RNP combinations containing amino acids 1 to 60 [i.e., 7_PB2_7_PB1_7_9(1–60)PA_7_NP_], 1 to 86 [i.e., 7_PB2_7_PB1_7_9(1–86)PA_7_NP_], and 1 to 120 [i.e., 7_PB2_7_PB1_7_9(1–120)PA_7_NP_] of CK/5(H9N2) PA were comparable to or lower than that of 7_PB2_7_PB1_7_PA_7_NP_ in HEK293T cells, whereas the RNP combination containing amino acids 1 to 191 of CK/5(H9N2) PA [i.e., 7_PB2_7_PB1_7_9(1–191)PA_7_NP_] possessed significantly higher activity (Fig. 2B). RT-qPCR analysis showed that the levels of all three species of viral RNA (i.e., vRNA, cRNA, and mRNA) were dramatically increased in A549 cells infected with the PG/S1421(H7N9) mutant bearing the 1–191 domain of CK/5(H9N2) PA (i.e., PG/S1421-CK/5PA_1–191_) relative to those of parental PG/S1421(H7N9) virus (Fig. 3), confirming that the 1–191 region of CK/5(H9N2) PA is essential for enhancing the transcription and replication of viral RNAs in human cells. When we tested the PG/S1421(H7N9)-backbone viruses with PAN chimeras in mice, we found that the three H7N9 chimeric PAN viruses with low polymerase activities (i.e., PG/S1421-CK/5PA_1–60_, PG/S1421-CK/5PA_1–86_, and PG/S1421-CK/5PA_1–120_) acquired the PB2 E627K mutation after one passage in mice (Fig. 2C). In contrast, PB2 627E was preserved during PG/S1421-CK/5PA_1–191_(H7N9) replication in mice (Fig. 2C). Recovered PG/S1421-CK/5PA_1–120_(H7N9) and PG/S1421-CK/5PA_1–191_(H7N9) viruses were passaged again in mice, and viruses recovered from the lungs were deep sequenced. The data showed that PG/S1421-CK/5PA_1–120_(H7N9) acquired the PB2 E627K mutation, whereas PG/S1421-CK/5PA_1–191_(H7N9) retained PB2 627E (Table S1). These results indicate that amino acid differences between PG/S1421(H7N9) and CK/5(H9N2) PA in region 121–191 are critical in determining the low polymerase activity of H7N9 AIVs and the emergence of the mammalian-adaptive PB2 E627K mutation.

The PA proteins of PG/S1421(H7N9) and CK/5(H9N2) differ by 12 amino acids in the PAN domain and by only four residues—142R, 147V, 171V, and 182L—in region 121–191 (Fig. S3). We found that residues 147V and 171V and different combinations of the four residues increased the polymerase activity in HEK293T cells in the background of the PG/S1421(H7N9) RNP complex, with the four residues combined increasing the polymerase activity the most (Fig. 2B). RT-qPCR analysis showed that the PG/S1421(H7N9) mutant bearing the four PA residues of CK/5(H9N2) (i.e., PG/S1421-PA_142R-147V-171V-182L_) grew in A549 cells with significantly higher levels of the three species of viral RNA than those of parental PG/S1421(H7N9) (Fig. 3). In mice, PG/S1421-PA_142R-147V-171V-182L_(H7N9) maintained PB2 627E, whereas the four PG/S1421(H7N9) mutant viruses bearing the individual CK/5(H9N2) PA residues acquired the PB2 E627K mutation during replication (Fig. 2C). We also found that three PG/S1421(H7N9) mutant viruses bearing three CK/5(H9N2) PA residues (i.e., PG/S1421-PA_142R-147V-171V_, PG/S1421-PA_142R-147V-182L_, and PG/S1421-PA_147V-171V-182L_) obtained the PB2 E627K mutation while replicating in mice, whereas a fourth triple-PA mutant PG/S1421(H7N9) virus (i.e., PG/S1421-PA_142R-171V-182L_) was not recovered from infected mouse lungs (Fig. 2C). Collectively, these results suggest that the replacement of the four PA residues (142K, 147I, 171I, and 182M) of PG/S1421(H7N9) with those of CK/5(H9N2) (142R, 147V, 171V, and 182L) may enhance the polymerase activity over a threshold capable of abolishing the need to acquire the PB2 E627K mutation in mammalian hosts. The role of these PA residues in determining the emergence of the PB2 E627K substitution was confirmed in the background of another H7N9 avian strain, A/chicken/Guangdong/SD008/2017 [CK/SD008(H7N9)] (18) (Table S2). Furthermore, we found that the four residues 142K, 147I, 171I, and 182M are highly conserved in the PA protein of different subtypes of AIVs (Table S3), which suggests that they may have broad effects in the adaptation of AIVs in mammals.

10.1128/mBio.01162-19.3 FIG S3 Amino acid differences between the N-terminal 1–252 domains (PANs) of PG/S1421(H7N9) and CK/5(H9N2). The positions of the 12 amino acids that differ between the PAN of PG/S1421(H7N9) and that of CK/5(H9N2) are shown. Download FIG S3, TIF file, 0.5 MB. Copyright © 2019 Liang et al. 2019 Liang et al. This content is distributed under the terms of the Creative Commons Attribution 4.0 International license.

10.1128/mBio.01162-19.9 TABLE S3 Analysis of the key PA residues that mediate the emergence of the PB2 E627K mutation in different subtypes of avian influenza virus. ^a^Sequences were derived from the GISAID EpiFlu database and the NCBI database and were analyzed by using the MAFFT multiple sequence alignment program (version 7). ^b^Percentage of PA proteins that contain all four PG/S1421(H7N9)-like PA residues, i.e., 142K, 147I, 171I, and 182M. ^c^Percentage of PA proteins that contain all four CK/5(H9N2)-like PA residues, i.e., 142R, 147V, 171V, and 182L. Download Table S3, PDF file, 0.1 MB. Copyright © 2019 Liang et al. 2019 Liang et al. This content is distributed under the terms of the Creative Commons Attribution 4.0 International license.

### H7N9 PA determines the sensitivity of PB2 627E polymerase to variations in human ANP32A expression.

**FIG 4:**
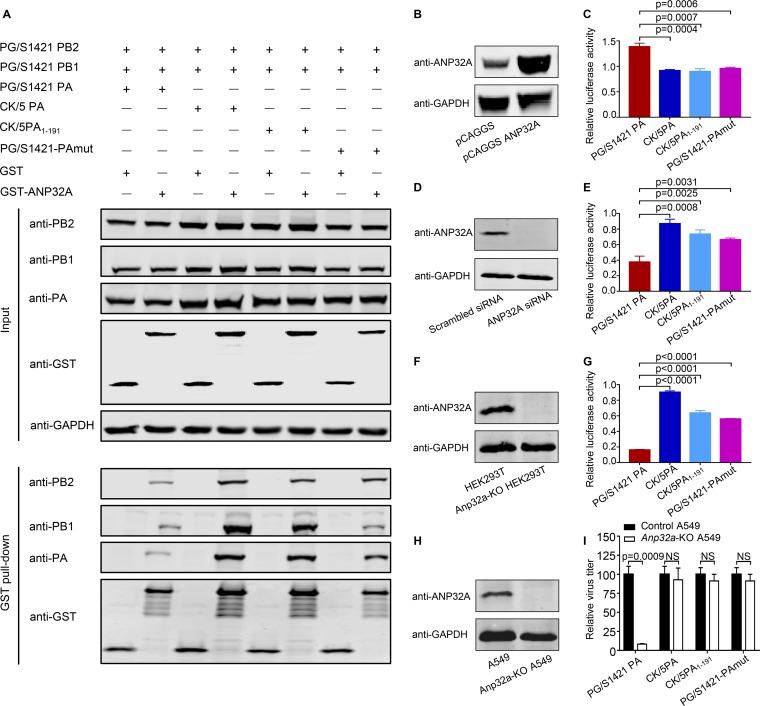
H7N9 PA determines the sensitivity of viral polymerase activity and growth to changes in human ANP32A expression. (A) GST pulldown assay to determine the interaction between human ANP32A and PG/S1421 (H7N9) polymerase complexes bearing different PAs. (B) ANP32A overexpression in HEK293T cells transfected with pCAGGS-ANP32A. (C) Luciferase assay in ANP32A-overexpressing cells (as shown in panel B) that were transfected with PG/S1421-PB2, -PB1, -NP, and different PAs, together with luciferase reporter constructs. Data shown are the ratio of the luciferase activity of the ANP32A-overexpressing group normalized to the pCAGGS control group. (D) siRNA knockdown of ANP32A in HEK293T cells. (E) Luciferase assay in ANP32A siRNA-treated HEK293T cells, as described for panel C. Data shown are the ratio of the luciferase activity of the ANP32A siRNA-treated group normalized to the scrambled siRNA-treated group. (F) Generation of *Anp32a*-KO HEK293T cells by using the CRISPR/Cas9 system. (G) Luciferase assay in *Anp32a*-KO HEK293T cells, as described for panel C. Data shown are the ratio of the luciferase activity of the *Anp32a*-KO HEK293T cells normalized to the HEK293T control group. (H) Generation of *Anp32a*-KO A549 cells by using the CRISPR/Cas9 system. (I) *Anp32a*-KO or control A549 cells were infected with the indicated PG/S1421(H7N9) viruses bearing different PAs. Data shown are the percentage of virus titers in *Anp32a*-KO cells normalized to the control A549 cells. CK/5PA_1-191_ stands for the construct PG/S1421-CK/5PA_1–191_; PG/S1421-PAmut stands for the construct PG/S1421-PA_142R-147V-171V-182L_; data are representative of three (C, E, and G) or two (I) independent experiments (means ± SDs); *P* values were determined by using a two-tailed unpaired Student's *t* test compared to the RNP complex bearing PG/S1421 PA (C, E, and G) and by using multiple *t* tests compared to viral titers in control A549 cells (I). NS, not significant.

Host factors ANP32A and ANP32B have been found to play an important role in viral genome transcription and replication of influenza virus (41–43). In comparison to ANP32B, ANP32A is reported to be involved in determining the host restriction of avian-like viral polymerase (PB2 627E) in human cells, demonstrating that avian ANP32A (avANP32A), due to unique sequence features, is able to effectively support the avian-like polymerase function compared with the human ANP32A (huANP32A) (41, 44, 45). However, whether huANP32A directly plays a role in the acquisition of the PB2 E627K substitution during the mammalian adaptation of AIVs has not been resolved. To address this point, we first determined the binding affinity of huANP32A for the trimeric polymerase complex of PG/S1421(H7N9) viruses bearing different PAs. To this end, we performed a GST pulldown assay in HEK293T cells that were transfected with a plasmid expressing GST or GST-ANP32A, together with plasmids for the expression of the viral polymerase (PG/S1421 PB2, PG/S1421 PB1, and different PAs). HuANP32A efficiently interacted with polymerase complexes containing CK/5(H9N2) PA, PG/S1421-CK/5PA_1–191_, or PG/S1421-PA_142R-147V-171V-182L_, whereas it bound less efficiently to the polymerase complex containing PG/S1421(H7N9) PA (Fig. 4A). We then tested the polymerase activity of different RNP complexes when huANP32A was overexpressed or knocked down. Interestingly, although the interaction between huANP32A and the polymerase complex containing PG/S1421(H7N9) PA was relatively weak, overexpression of huANP32A in HEK293T cells (Fig. 4B) enhanced the polymerase activity of the PG/S1421(H7N9) RNP complex (by approximately 1.4-fold) (Fig. 4C). In contrast, the polymerase activities of the RNP complexes bearing CK/5(H9N2) PA, PG/S1421-CK/5PA_1–191_, or PG/S1421-PA_142R-147V-171V-182L_ were relatively unaffected by huANP32A overexpression (Fig. 4C). We also knocked down huANP32A expression by using short interfering RNA (siRNA) in HEK293T cells (Fig. 4D and Fig. S4A). Significantly, knockdown of huANP32A expression led to an over 60% reduction in the polymerase activity of the PG/S1421(H7N9) RNP complex, whereas the polymerase activities of PG/S1421(H7N9) RNPs bearing CK/5(H9N2) PA, PG/S1421-CK/5PA_1–191_, or PG/S1421-PA_142R-147V-171V-182L_ were much less affected by huANP32A knockdown (Fig. 4E). These results demonstrate that the diversity among different PAs influences the sensitivity of the H7N9 PB2 627E polymerase to up- or downregulation of human ANP32A expression in human cells. Furthermore, the H7N9 PA protein is responsible for the low activity of PB2 627E polymerase, being highly sensitive to variations in human ANP32A expression.

10.1128/mBio.01162-19.4 FIG S4 Effect of siRNA knockdown of ANP32A or CRISPR/Cas9 knockout of ANP32A on cell viability. (A) Cell viability of siRNA-treated HEK293T cells. (B and C) Cell viability of ANP32A-knockout HEK293T (B) or A549 (C) cells. Cell viability was measured by using a CellTiter-Glo assay. The data are presented as means ± SDs for triplicates of a representative experiment. NS, not significant. Data are representative of three independent experiments (A to C). Download FIG S4, TIF file, 0.6 MB. Copyright © 2019 Liang et al. 2019 Liang et al. This content is distributed under the terms of the Creative Commons Attribution 4.0 International license.

To further confirm the role of huANP32A in modulating the polymerase activity of different PG/S1421(H7N9) RNP complexes, we generated an *Anp32a*-knockout (KO) HEK293T cell line by using the CRISPR/Cas9 system (46) (Fig. 4F and Fig. S4B). Strikingly, the polymerase activity of the PG/S1421(H7N9) RNP complex was reduced to only 16% in *Anp32a*-KO cells compared with control cells, whereas the activity of H7N9 RNP complexes bearing CK/5(H9N2) PA, PG/S1421-CK/5PA_1–191_, or PG/S1421-PA_142R-147V-171V-182L_ in *Anp32a*-KO cells retained levels of 56% to 90% of those of the control cells (Fig. 4G). We also quantified the yield of infectious H7N9 viruses from *Anp32a*-KO and control A549 cells. Upon knockout of *Anp32a* (Fig. 4H and Fig. S4C), the virus titer of PG/S1421(H7N9) decreased to less than 10% of that of the control cells (Fig. 4I). In contrast, the titers of PG/S1421-CK/5PA(H7N9), PG/S1421-CK/5PA_1–191_(H7N9), and PG/S1421-PA_142R-147V-171V-182L_(H7N9) decreased only slightly in the absence of huANP32A (Fig. 4I). Together, these data confirm that the H7N9 PA protein is responsible for the vulnerability of the polymerase activity and growth of PB2 627E virus to the depletion of human ANP32A protein.

We also performed a minigenome assay for the PG/S1421(H7N9) RNP bearing the PB2 627K mutation and found that its polymerase activity was unchanged when the expression of huANP32A was knocked out (Fig. S5A). Moreover, the depletion of ANP32A protein did not have any adverse effects on the growth of PB2 627K mutant virus in A549 cells (Fig. S5B). These results indicate that once the viral polymerase activity is compensated for by the PB2 E627K mutation, the polymerase function and virus growth are no longer influenced by huANP32A in human cells.

10.1128/mBio.01162-19.5 FIG S5 Effect of *Anp32a* knockout on the polymerase activity and virus growth of PG/S1421(H7N9) and PG/S1421(H7N9) PB2 627K mutant. (A) Relative polymerase activity in *Anp32a*-KO or control HEK293T cells. Data shown are the ratio of the luciferase activity in *Anp32a*-KO HEK293T cells normalized to the HEK293T control group. (B) Titers of PG/S1421 and PG/S1421 PB2 627K mutant in *Anp32a*-KO or control A549 cells. *P* values were determined by using a two-tailed unpaired Student's *t* test. NS, not significant. Data are representative of three (A) or two (B) independent experiments (means ± SDs). Download FIG S5, TIF file, 0.7 MB. Copyright © 2019 Liang et al. 2019 Liang et al. This content is distributed under the terms of the Creative Commons Attribution 4.0 International license.

### The impaired H7N9 PB2 627E polymerase activity incurred by ANP32A depletion drives the acquisition of adaptive PB2 mutation.

**FIG 5:**
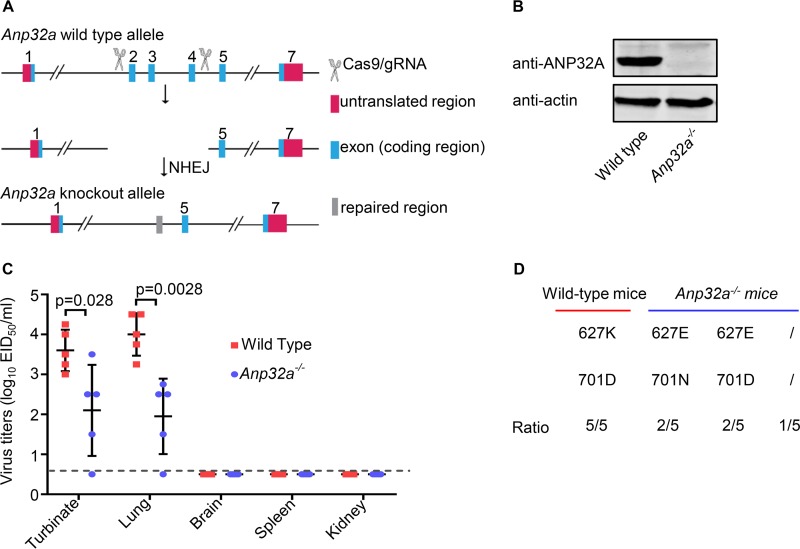
PG/S1421(H7N9) virus replication is impaired by ANP32A depletion in *Anp32a^−/−^* mice, driving the emergence of the PB2 D701N mutation instead of E627K. (A) Schematic illustration of the strategy used to generate *Anp32a^−/−^* mice. Two sgRNAs were designed to delete exons 2 to 4 of the *Anp32a* gene. The genotype of the generated mice was identified by PCR and sequencing. (B) Knockout of the *Anp32a* gene was verified by Western blotting of mouse lung homogenates with an anti-ANP32A MAb. (C) Replication of PG/S1421(H7N9) in *Anp32a^−/−^* mice. Wild-type (WT) and *Anp32a^−/−^* C57BL/6J mice (*n* = 5 per genotype) were inoculated with 10^6^ EID_50_ of PG/S1421(H7N9). Organs were collected on day 5 p.i. and titrated for virus infectivity in eggs. The data are presented as means ± SDs for organ samples of five mice. *P* values were determined by using multiple *t* tests (one unpaired *t* test per row). (D) Phenotype of PB2 residues 627 and 701 during replication of PG/S1421(H7N9) virus in WT or *Anp32a^−/−^* mice (*n* = 5). Viral RNAs were isolated from lung homogenates of infected mice on day 5 p.i., and the PB2 segment was amplified by RT-PCR and sequenced to monitor adaptive mutations arising during passage in mice. /, virus was not detected in one of the five *Anp32a^−/−^* mice.

We demonstrated that the polymerase activity and growth of PG/S1421(H7N9) are dramatically impaired by the depletion of ANP32A protein in human cells. To determine how the PG/S1421(H7N9) virus behaves under such conditions *in vivo*, we generated *Anp32a^−/−^* C57BL/6J mice by use of CRISPR/Cas9-mediated gene targeting. A targeting construct was designed to delete exons 2 to 4 of *Anp32a* (Fig. 5A). Deletion of *Anp32a* was confirmed by sequencing, and the absence of ANP32A protein from *Anp32a^−/−^* mice was confirmed by Western blotting of lung extracts (Fig. 5B). The *Anp32a^−/−^* mice and matched wild-type (WT) controls were intranasally inoculated with PG/S1421(H7N9), and mouse lungs were collected on day 5 p.i. for virus isolation. The titers of PG/S1421(H7N9) virus in *Anp32a^−/−^* mice were dramatically decreased compared with those in WT mice (Fig. 5C). Sequence analysis confirmed that the PB2 E627K mutation was acquired by virus in the lungs of the infected WT mice (Fig. 5D). Notably, PG/S1421(H7N9) replication was not detected in one of the five *Anp32a^−/−^* mice, PB2 627E was maintained in viruses recovered from two *Anp32a^−/−^* mice, and a PB2 D701N mutation emerged in viruses in the other two *Anp32a^−/−^* mice (Fig. 5D). These results indicate that the impaired polymerase activity caused by the depletion of ANP32A protein significantly reduced the replication of H7N9 PB2 627E virus in *Anp32a^−/−^* mice. The absence of ANP32A protein abolished the acquisition of the PB2 E627K mutation and instead forced the virus down an alternative adaptive pathway to acquire the compensatory PB2 D701N mutation.

## DISCUSSION

Introduction of adaptive mutations in viral polymerase proteins is critical for AIVs to cross the species barrier to infect and kill humans. The role of the PB2 E627K mutation in mammalian adaptation has been well established for a wide range of AIVs, such as the H5N1, H7N7, H7N9, and H10N8 subtypes (7, 22, 47, 48). In this study, we used the H7N9 virus model, which easily acquires the PB2 E627K mutation when it infects humans (18), to investigate the molecular basis for the emergence of PB2 E627K during human adaptation. Our results show that the polymerase activity of PG/S1421(H7N9) is significantly lower than that of CK/5(H9N2) in HEK293T cells, which is the opposite of our findings in avian DF-1 cells. Importantly, we found that the low polymerase activity attributed to the PA protein of H7N9 AIVs in human cells is the intrinsic driving force to acquire the PB2 E627K mutation during replication in wild-type mice. Moreover, the H7N9 PA protein is responsible for the sensitivity of the viral polymerase activity and growth to variations in the expression of the huANP32A protein in human cells. The impaired polymerase activity of H7N9 AIV due to the knockout of ANP32A reduces virus replication in *Anp32a^−/−^* mice. Under such conditions, ANP32A depletion abolishes the acquisition of the PB2 E627K mutation, thereby forcing the virus to acquire the alternative adaptive PB2 D701N mutation. Our data thus reveal that the prominent ability of H7N9 AIVs to acquire the mammalian-adaptive PB2 E627K mutation is driven by the intrinsic low polymerase activity attributed to the viral PA protein, which also involves interplay with mammalian ANP32A.

The PA protein has been well documented to be involved in the pathogenesis of influenza virus (32–36). It is separated into two domains linked by a protease-sensitive peptide (49). The crystal structure of the N-terminal PA domain has been resolved for several influenza viruses (50, 51). The amino acid position 142 is correlated with the pathogenicity of AIV (52, 53) and is part of a loop connecting α5 and β4, residue 147 is a part of sheet β4, and residues 171 and 182 are localized at helix α6. To better view the location of these four critical PA residues, we mapped them in the structure of the viral polymerase complex (54) (see Fig. S6 in the supplemental material). Although these four PA residues are not structurally adjacent to the PB2 627 residue, they could possibly affect the interaction of PA with other polymerase subunits and/or host proteins. By examining these four PA residues in AIVs of subtypes H1 through H16, we found that most AIVs possess the PG/S1421(H7N9)-like constellation (i.e., 142K, 147I, 171I, and 182M) and that few H9 AIVs (0.12%) had the CK/5(H9N2)-like constellation (i.e., 142R, 147V, 171V, and 182L) (Table S3). Of note, the four PG/S1421(H7N9)-like PA residues are primarily responsible for the low viral polymerase activity in human cells. Acquiring the potent polymerase substitution PB2 E627K is an effective way to overcome the species barrier.

10.1128/mBio.01162-19.6 FIG S6 Location of four PA residues, 142, 147, 171, and 182, and the PB2 627 residue on the three-dimensional (3D) structure of influenza A virus polymerase (PDB 4WSB). The PB2, PB1, and PA subunits are shown in violet, blue, and green, respectively. Download FIG S6, TIF file, 2.2 MB. Copyright © 2019 Liang et al. 2019 Liang et al. This content is distributed under the terms of the Creative Commons Attribution 4.0 International license.

A previous study by Long et al. focused on how the avian polymerase is restricted in human cells and identified that avANP32A can strongly support PB2 627E polymerase activity because it possesses an additional 33 amino acids compared with huANP32A (41). Domingues and Hale identified a SUMO-interacting-motif-like sequence unique to avANP32A that promotes PB2 627E polymerase activity (45). Baker et al. further demonstrated that avANP32A restores RNP complex assembly for avian polymerase in human cells by enhancing RNA synthesis (44). However, these studies did not explore the role of huANP32A in the emergence of the PB2 E627K mutation during the replication of AIVs under unfavorable conditions in human cells. In the present study, we found that H7N9 PB2 627E polymerases bearing different PAs show diversity in their sensitivity to the up- or downregulation of huANP32A expression. When PG/S1421(H7N9) PA was replaced with CK/5(H9N2) PA, PG/S1421-CK/5PA_1–191_, or PG/S1421-PA_142R-147V-171V-182L_, the polymerase activity increased, abrogating the need to acquire the PB2 E627K mutation during virus replication in mice. The increased polymerase activity was accompanied by reduced sensitivity to changes in huANP32A expression even though the interaction between the polymerase and huANP32A was enhanced. In contrast, the low polymerase activity of PG/S1421(H7N9) conferred by the viral PA protein was highly sensitive to the depletion of huANP32A protein although their interaction was comparatively weak, suggesting that the H7N9 PB2 627E polymerase can efficiently exploit human ANP32A. The impaired PB2 627E polymerase activity due to the depletion of ANP32A led to reduced virus replication and created the need for adaptive mutations in *Anp32a^−/−^* mice. Moreover, the depletion of ANP32A abolished the acquisition of the adaptive PB2 E627K mutation, forcing the virus to use an alternative adaptive pathway to acquire the PB2 D701N mutation.

The activity of PG/S1421(H7N9) polymerase bearing the PB2 627K mutation was not affected by the depletion of ANP32A protein in human cells. The replication of the PB2 627K mutant virus was also unchanged in this context. These findings imply that huANP32A has less of a role in maintaining the high polymerase activity of H7N9 PB2 627K virus. The enhanced polymerase activity of H7N9 PB2 627K virus is most likely mainly conferred by the attribute of the PB2 627K residue, which forms a contiguous basic face in the PB2 627 domain that is important for RNA binding and polymerase function in mammalian cells (55).

The introduction of CK/5(H9N2) PB1 into the PG/S1421(H7N9) backbone led to the appearance of the adaptive mutation PB2 D701N (9, 10, 38–40), during the replication of PG/S1421-CK/5PB1(H7N9) in mice. This finding suggests that the acquisition of the PB2 D701N mutation may correlate with the origin of the viral PB1 gene. Furthermore, ANP32A depletion diverts the H7N9 polymerase adaptation from acquiring the PB2 E627K mutation, instead forcing the polymerase adaptation into an alternative pathway of acquiring PB2 D701N. Our data suggest that the host factors involved in the adaptation of the avian polymerase may not be exclusive. Previous studies have shown that the PB2 D701N mutation is responsible for enhanced binding with importin α, leading to increased virus replication in mammalian cells (56, 57). However, whether importin α is involved in the emergence of the PB2 D701N mutation in the background of H7N9 viruses remains to be further investigated.

In summary, our study provides new insights into the biological mechanism employed by H7N9 AIVs to acquire the PB2 E627K substitution in order to adapt and become pathogenic in humans. We discovered that the low polymerase activity of H7N9 AIVs in human cells, an attribute conferred by the viral PA protein, is the intrinsic driving force in the emergence of the adaptive PB2 E627K mutation, which also involves mammalian ANP32A. The findings thus enhance our understanding of the adaptation and pathogenesis of AIVs in humans and other mammals.

## MATERIALS AND METHODS

### Biosafety statement and facility.

All experiments with live H7N9 viruses were conducted within the enhanced animal biosafety level 3 (ABSL3+) facility at the Harbin Veterinary Research Institute (HVRI) of the Chinese Academy of Agricultural Sciences (CAAS) approved for such use by the Ministry of Agriculture and Rural Affairs of the People’s Republic of China. All animal studies were approved by the Review Board of the HVRI, CAAS. The details of the facility and the biosafety and biosecurity measures used have been previously reported (25, 58).

### Cells and viruses.

HEK293T cells and chicken fibroblast cells (DF-1) were cultured in DMEM (Life Technologies) supplemented with 10% fetal bovine serum (Sigma-Aldrich), human lung carcinoma cells (A549) were cultured in F12K medium (Life Technologies) supplemented with 10% FBS, and Madin-Darby canine kidney (MDCK) cells were cultured in MEM (Life Technologies) containing 5% newborn calf serum (Sigma-Aldrich). All cells were maintained in a humidified incubator containing 5% CO_2_ at 37°C (HEK293T, A549, and MDCK cells) or 39°C (DF-1 cells). The five H9N2 strains used, A/chicken/Henan/5/1998 [CK/5(H9N2)], A/chicken/Guangxi/9/1999 [CK/9(H9N2)], A/chicken/Guangxi/C2163/2012 [CK/C2163(H9N2)], A/chicken/Shanghai/SC197/2013 [CK/SC197(H9N2)], and A/chicken/Zhejiang/SC324/2013 [CK/SC324(H9N2)] were isolated between 1998 and 2013 and reported previously (29, 30). The H7N9 viruses A/pigeon/Shanghai/S1421/2013 [PG/S1421(H7N9)] and A/chicken/Guangdong/SD008/2017 [CK/SD008(H7N9)] were reported in previous studies (18, 19). All H7N9 and H9N2 viruses were propagated in embryonated chicken eggs.

### Plasmids.

The construction of plasmids for virus rescue was performed as described previously (31). The PB2, PB1, PA, and NP genes of the respective influenza viruses, as well as human ANP32A (huANP32A), were cloned into the mammalian expression vector pCAGGS. GST-tagged ANP32A was constructed in pCAGGS with a GST tag at the N terminus and an SV40 large-T antigen nuclear localization signal (NLS) at the C terminus. Chimeric PA genes, single or multiple point mutants of the PA genes, and the PB2 E627K mutant (generated by using a PCR approach) were cloned into vectors pCAGGS and pHH21. Plasmid pHH21-SC09NS F-Luc and paviPolI-T-Luc, for the expression of a virus-like RNA bearing the firefly luciferase gene under the control of the human or avian RNA polymerase I promoter, were reported previously (34, 37). All constructs were sequenced to ensure the absence of unwanted mutations. The primer sequences used for cloning are available upon request.

### Antibodies.

The following primary antibodies were purchased from commercial sources: rabbit anti-PB2 polyclonal antibody (PAb) (GeneTex), rabbit anti-PB1 PAb (GeneTex), rabbit anti-PA PAb (GeneTex), rabbit anti-ANP32A monoclonal antibody (MAb) (Cell Signaling Technology), rabbit anti-GAPDH PAb (Proteintech), mouse anti-actin MAb (Santa Cruz), and mouse anti-GST MAb (GenScript Biotech). The secondary antibodies DyLight 800 goat anti-mouse IgG(H+L) and DyLight 800 goat anti-rabbit IgG(H+L) (KPL) were used for Western blotting.

### Passage of H9N2 and H7N9 viruses in MDCK cells.

Confluent MDCK cells were inoculated for 1 h with the indicated H9N2 and H7N9 viruses at a multiplicity of infection (MOI) of 0.01. The cells were then washed and maintained in 1× MEM (0.3% BSA, 0.5 μg/ml *N*-tosyl-l-phenylalanyl chloromethyl ketone [TPCK]-treated trypsin) at 37°C. After 48 h of incubation, the virus supernatants were collected for sequencing and the next passage in MDCK cells.

### Generation of reassortant viruses by reverse genetics.

Reassortant viruses were generated by using the reverse genetics system as described previously (31). The rescued viruses were detected by using a hemagglutination assay and were fully sequenced to ensure the absence of unwanted mutations.

### Dual-luciferase reporter assay.

HEK293T cells and siRNA-treated or *Anp32a*-KO (see below) HEK293T cells were transfected with pCAGGS constructs expressing viral PB2, PB1, PA (parental or mutant), and NP genes (0.5 μg each); the construct pHH21-SC09NS F-Luc (0.1 μg); and an internal control, pRL-TK (50 ng; Promega), by using Lipofectamine LTX and Plus reagents (Invitrogen). DF-1 cells were similarly transfected by using TransIT-X2 (Mirus) except for the inclusion of paviPolI-T-Luc instead of pHH21-SC09NS F-Luc. Cells were incubated at 37°C (HEK293T cells) or 39°C (DF-1 cells) for 30 h, and cell lysates were subsequently prepared by using the dual-luciferase reporter assay system (Promega). The luciferase activities were measured on a GloMax 96 microplate luminometer (Promega).

### Quantification of viral RNA species.

A549 cells grown in 6-well plates were infected with the indicated viruses at an MOI of 2. Total RNA was extracted by using an RNeasy Plus Mini Kit (Qiagen) at 6 and 8 h postinfection (p.i.) according to the manufacturer’s instructions. Relative quantities of viral NP genomic RNA (vRNA), cRNA, and mRNA were determined by RT-qPCR using a tagged-primer system as described previously (37).

### GST pulldown assay.

HEK293T cells grown in 10-cm dishes were transfected with the indicated plasmids (PG/S1421 PB2 and PG/S1421 PB1, 10 μg each; different PAs, 4 μg each; GST or GST-ANP32A, 6 μg) by using the Lipofectamine LTX and Plus reagents. At 48 h posttransfection, cells were lysed with NP-40 buffer (Beyotime Biotechnology). After centrifugation, the cleared lysates were incubated with glutathione-Sepharose 4B resin (GE Healthcare) at 4°C for 3 h. After three washes with cold PBS, the resin-bound proteins were separated by SDS-PAGE and detected by using a standard Western blotting procedure.

### siRNA knockdown.

AllStars negative-control siRNA (1027281) or huANP32A FlexiTube siRNA (SI02655212) (Qiagen) at a concentration of 30 nM was transfected into HEK293T cells seeded in 24-well plates by using the Lipofectamine RNAiMAX transfection reagent (Invitrogen). At 48 h posttransfection, the siRNA knockdown efficiency was confirmed by Western blotting, and the luciferase assay was performed in siRNA-treated cells as described above.

### Generation of *Anp32a*-KO HEK293T cells and *Anp32a*-KO A549 cells.

*Anp32a*-knockout HEK293T and A549 cells were generated by using the CRISPR/Cas9 system. HEK293T cells grown in a 10-cm dish were transfected with 10 μg of pSpCas9(BB)-2A-GFP (pX458) containing a target sequence (5′-CTTTGGTAAGTTTGCGATTG-3′) complementary to exon 2 of human *Anp32a* by using TransIT-LT1 (Mirus). For A549 cells, 1 × 10^6^ cells were electroporated using the Neon transfection system 100-μl kit (Thermo Fisher Scientific) according to the manufacturer’s instructions. At 48 h posttransfection, cells were trypsinized and sorted into 96-well plates at 1 cell/well using fluorescence-activated cell sorting (FACS) with a FACS-Aria II cell sorter (BD BioSciences). GFP-expressing cells were expanded to obtain individual clones. The knockout of HuANP32A expression was confirmed by Western blotting using an anti-ANP32A antibody (15491; Cell Signaling Technology).

The luciferase assay in *Anp32a*-knockout HEK293T cells was performed as described above.

To study the effect of *Anp32a* knockout on the growth of parental or mutant PG/S1421(H7N9) viruses, *Anp32a*-KO A549 cells were infected with the indicated virus at an MOI of 0.1. Supernatants were collected at 24 h p.i., and virus titers were determined in embryonated chicken eggs.

### Cell viability assay.

Cell viability was determined by using the CellTiter-Glo kit (Promega) as described previously (37, 59). Briefly, HEK293T cells seeded in opaque-walled 96-well plates were transfected with siRNA targeting ANP32A or with AllStars negative-control siRNA at a concentration of 30 nM. At 48 h posttransfection, luminescence was measured with the CellTiter-Glo kit (Promega).

The cell viability assay was similarly performed for the *Anp32a*-KO HEK293T cells, *Anp32a*-KO A549 cells, and the corresponding control cells grown in opaque-walled 96-well plates.

### Generation of the *Anp32a*-knockout mouse model.

The *Anp32a*-knockout mouse model used in this study was designed and developed by Shanghai Model Organisms Center, Inc. Briefly, Cas9 mRNA was *in vitro* transcribed with the mMessage mMachine T7 Ultra kit (Ambion) according to the manufacturer’s instructions. Two single guide RNAs (sgRNAs), 5′-GTGAGCAGGGACTGAGGTAGCGG-3′ and 5′-ACACCATACTGTGTGCAACAGGG-3′, were targeted to delete exons 2 to 4 of the *Anp32a* gene. The sgRNAs were *in vitro* transcribed using the MEGAshortscript kit (Thermo Fisher Scientific). The *in vitro*-transcribed Cas9 mRNA and sgRNAs were then injected into zygotes of C57BL/6J mice and transferred to pseudopregnant recipients. The obtained F0 mice were validated by PCR and sequencing. The positive F0 mice were crossed with C57BL/6J mice to obtain F1 heterozygous *Anp32a*-knockout mice. The genotype of the F1 mice was determined by PCR and confirmed by sequencing. Male and female F1 heterozygous mice were intercrossed to produce homozygous *Anp32a*-knockout mice.

### Animal experiments.

Six-week-old female BALB/c mice (Vital River Laboratories, China) were lightly anesthetized with CO_2_ and intranasally infected with 10^6^ 50% egg infective doses (EID_50_) of the indicated H9N2 viruses or parental, reassortant, or mutant H7N9 viruses. On day 5 p.i., three mice per group were euthanized, and lung samples were collected and used for sequencing, virus titration in eggs, or the next passage in mice.

Six-week-old female wild-type (WT) or *Anp32a*-knockout C57BL/6J mice (see above) were intranasally inoculated with 10^6^ EID_50_ of PG/S1421(H7N9). On day 5 p.i., five mice per group were euthanized, and organs (including lungs, nasal turbinates, brains, spleens, and kidneys) were collected for virus titration and sequencing.

### Deep sequencing.

Viral RNA was extracted from lung homogenates with the QIAamp Viral RNA Mini Kit (Qiagen) and reverse transcribed into cDNA by use of Uni12 primer (5′-AGCRAAAGCAGG-3′). The entire genome of the viruses was amplified with the inﬂuenza A virus-speciﬁc primers MBTuni-12 and MBTuni-13 (60). Next-generation sequencing libraries were constructed by using the TruSeq Nano DNA library prep kit (Illumina). For each sample, 100 ng of DNA was randomly fragmented to <500 bp by sonication. The fragments were treated with End Repair Mix for end repairing and with A-Tailing Mix for dA tailing, followed by a T-A ligation to add adaptors to both ends. Each sample was then amplified by PCR using P5 and P7 primers, with both primers carrying sequences that could anneal with the flow cell to perform bridge PCR and the P7 primer carrying a six-base index allowing for multiplexing. The PCR products were cleaned using SPB beads, validated using an Agilent 2100 Bioanalyzer (Agilent Technologies), and quantified by using a Qubit2.0 fluorometer (Invitrogen). Then, libraries with different indices were multiplexed and loaded onto an Illumina HiSeq instrument. Sequencing was carried out using a 2 × 150 paired-end configuration; image analysis and base calling were conducted by the HiSeq Control Software (HCS) + OLB + GAPipeline-1.6 (Illumina) on the HiSeq instrument. The genomes of all samples sequenced yielded more than 5,000-fold genome coverage depth (with raw sequencing data of approximately 2.0 Gb obtained per sample). The sequences were processed and analyzed by Genewiz (Genewiz Biotechnology).

### Statistical analysis.

Quantitative data are presented as means ± SDs from at least three biological replicates. Data were statistically analyzed with a two-tailed unpaired Student's *t* test or multiple *t* tests by using GraphPad Prism 7.0 software. Statistical parameters are reported in the figures and figure legends. *P* values of <0.05 were considered statistically significant.

### Data availability.

The data generated or analyzed during this study are included in this paper. The nucleotide sequences of the H7N9 and H9N2 viruses used in this study have been deposited in GenBank [accession numbers DQ064559, DQ064532, DQ064505, DQ064370, DQ064451, DQ064424, DQ064397, and DQ064478 for CK/5(H9N2); DQ064553, DQ064526, DQ064499, DQ064364, DQ064445, DQ064418, DQ064391, and DQ064472 for CK/9(H9N2); KM113194 to KM113201 for CK/C2163(H9N2); KM113066 to KM113073 for CK/SC197(H9N2); KM113058 to KM113065 for CK/SC324(H9N2); CY147177 to CY147184 for PG/S1421(H7N9); and MF630034 to MF630041 for CK/SD008(H7N9)]. The raw deep-sequencing data are available from the corresponding author upon reasonable request.
