# Supplementary material for: Low Polymerase Activity Attributed to PA Drives the Acquisition of the PB2 E627K Mutation of H7N9 Avian Influenza Virus in Mammals
Source: mBio. 2019 Jun 18;10(3):e01162-19. doi: 10.1128/mBio.01162-19 (PMC6581862; doi:10.1128/mBio.01162-19)
Supplement: TABLE S1 [file mBio.01162-19-st001.pdf]

Table S1

Sample	Frequency of the indicated amino acid at key adaptive positions in PB2 (%)[a]									
	271		588		591		627		701	
	T	A	A	V	Q	K	E	K	D	N
PG/S1421(H7N9) stock virus	100	0[b]	100	0	100	0	100	0	100	0
PG/S1421-CK/5PA(H7N9) stock virus	100	0	100	0	100	0	100	0	100	0
PG/S1421-CK/5PA(H7N9) P2-1	100	0	100	0	100	0	100	0	100	0
PG/S1421-CK/5PA(H7N9) P2-2	100	0	100	0	100	0	100	0	100	0
PG/S1421-CK/5PA(H7N9) P2-3	100	0	100	0	100	0	100	0	100	0
PG/S1421-CK/5PA$_{1-120}$(H7N9) stock virus	100	0	100	0	100	0	100	0	100	0
PG/S1421-CK/5PA$_{1-120}$(H7N9) P2-1	100	0	100	0	100	0	0.68	99.32	100	0
PG/S1421-CK/5PA$_{1-120}$(H7N9) P2-2	100	0	100	0	100	0	1.28	98.72	100	0
PG/S1421-CK/5PA$_{1-120}$(H7N9) P2-3	100	0	100	0	100	0	0.27	99.73	100	0
PG/S1421-CK/5PA$_{1-191}$(H7N9) stock virus	100	0	100	0	100	0	100	0	100	0
PG/S1421-CK/5PA$_{1-191}$(H7N9) P2-1	100	0	100	0	100	0	100	0	100	0
PG/S1421-CK/5PA$_{1-191}$(H7N9) P2-2	100	0	100	0	100	0	100	0	100	0
PG/S1421-CK/5PA$_{1-191}$(H7N9) P2-3	100	0	100	0	100	0	100	0	100	0
PG/S1421-PA$_{142R-147V-171V-182L}$(H7N9) stock virus	100	0	100	0	100	0	100	0	100	0
PG/S1421-PA$_{142R-147V-171V-182L}$(H7N9) P2-1	100	0	100	0	100	0	100	0	100	0
PG/S1421-PA$_{142R-147V-171V-182L}$(H7N9) P2-2	100	0	100	0	100	0	100	0	100	0
PG/S1421-PA$_{142R-147V-171V-182L}$(H7N9) P2-3	100	0	100	0	100	0	100	0	100	0
