# Supplementary figures and images for: Low Polymerase Activity Attributed to PA Drives the Acquisition of the PB2 E627K Mutation of H7N9 Avian Influenza Virus in Mammals
Source: mBio. 2019 Jun 18;10(3):e01162-19. doi: 10.1128/mBio.01162-19 (PMC6581862; doi:10.1128/mBio.01162-19)

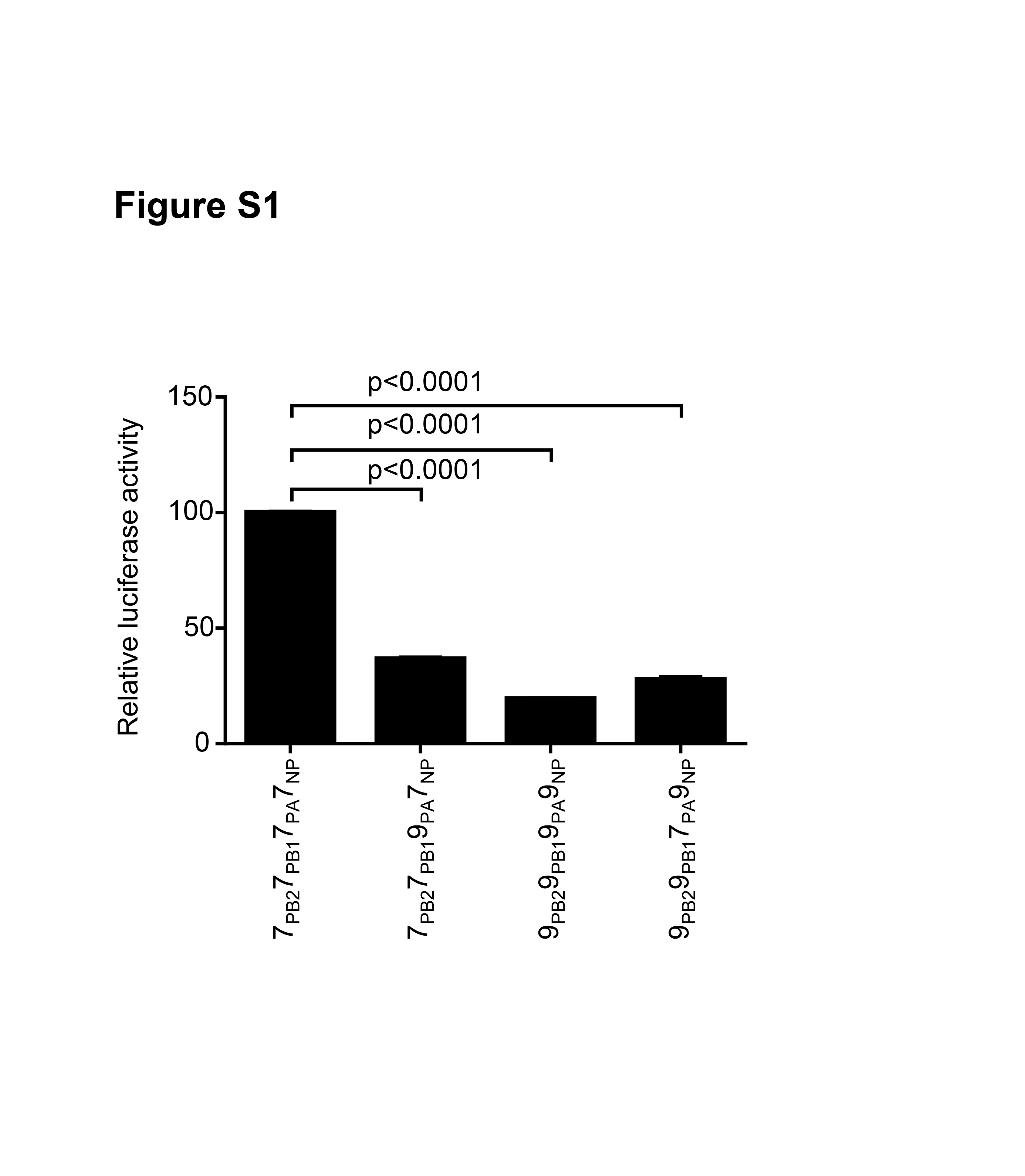

Supplement: FIG S1 [file mBio.01162-19-sf001.tif]

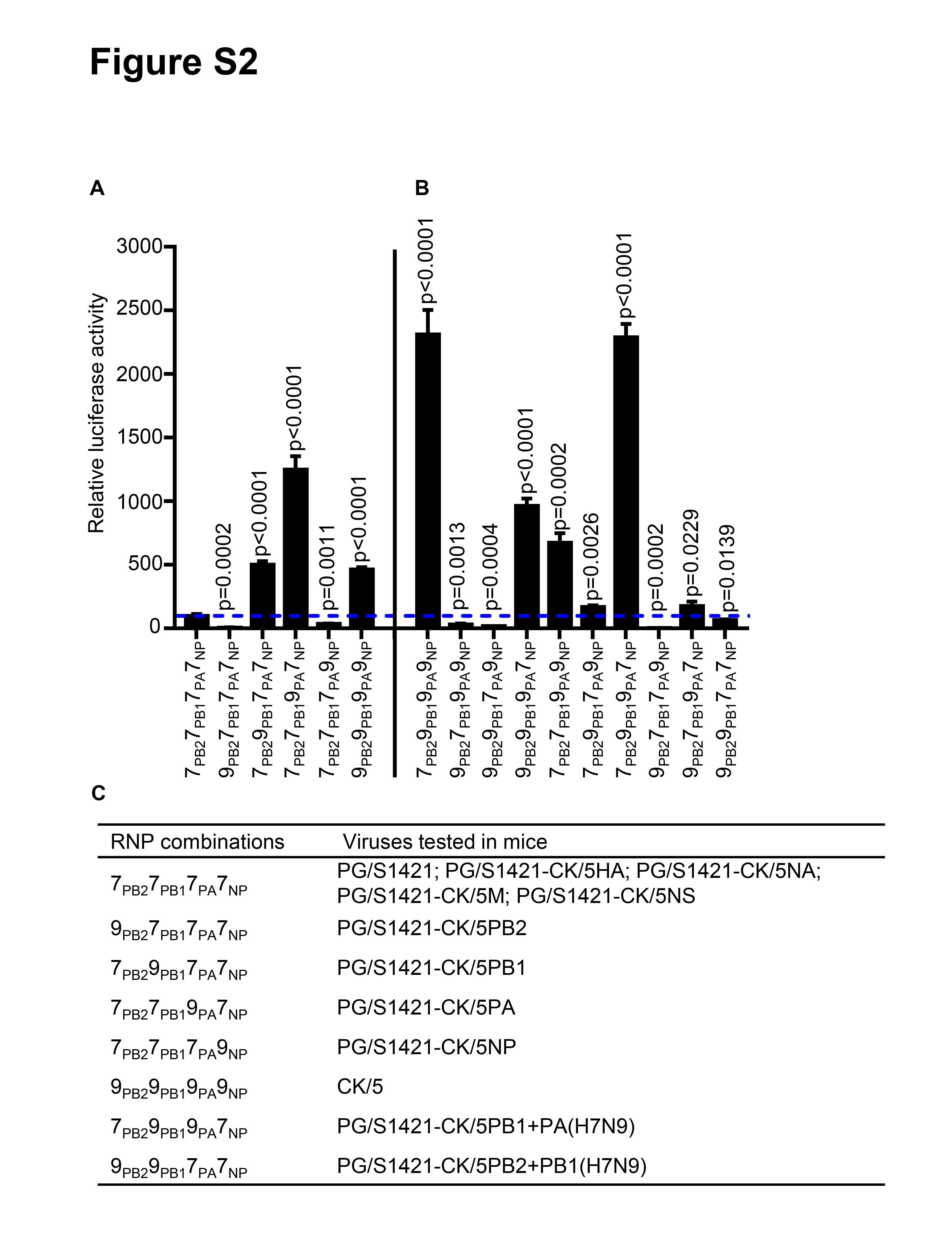

Supplement: FIG S2 [file mBio.01162-19-sf002.tif]

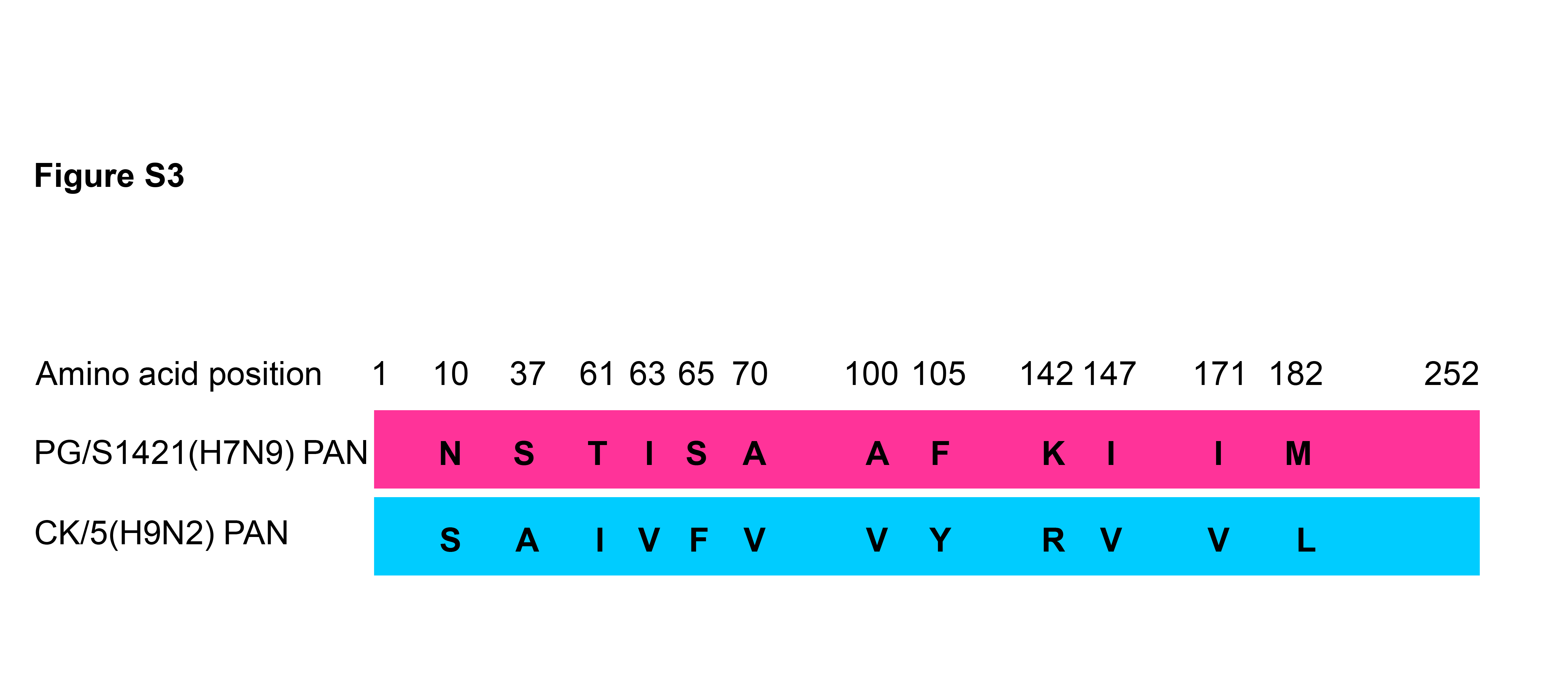

Supplement: FIG S3 [file mBio.01162-19-sf003.tif]

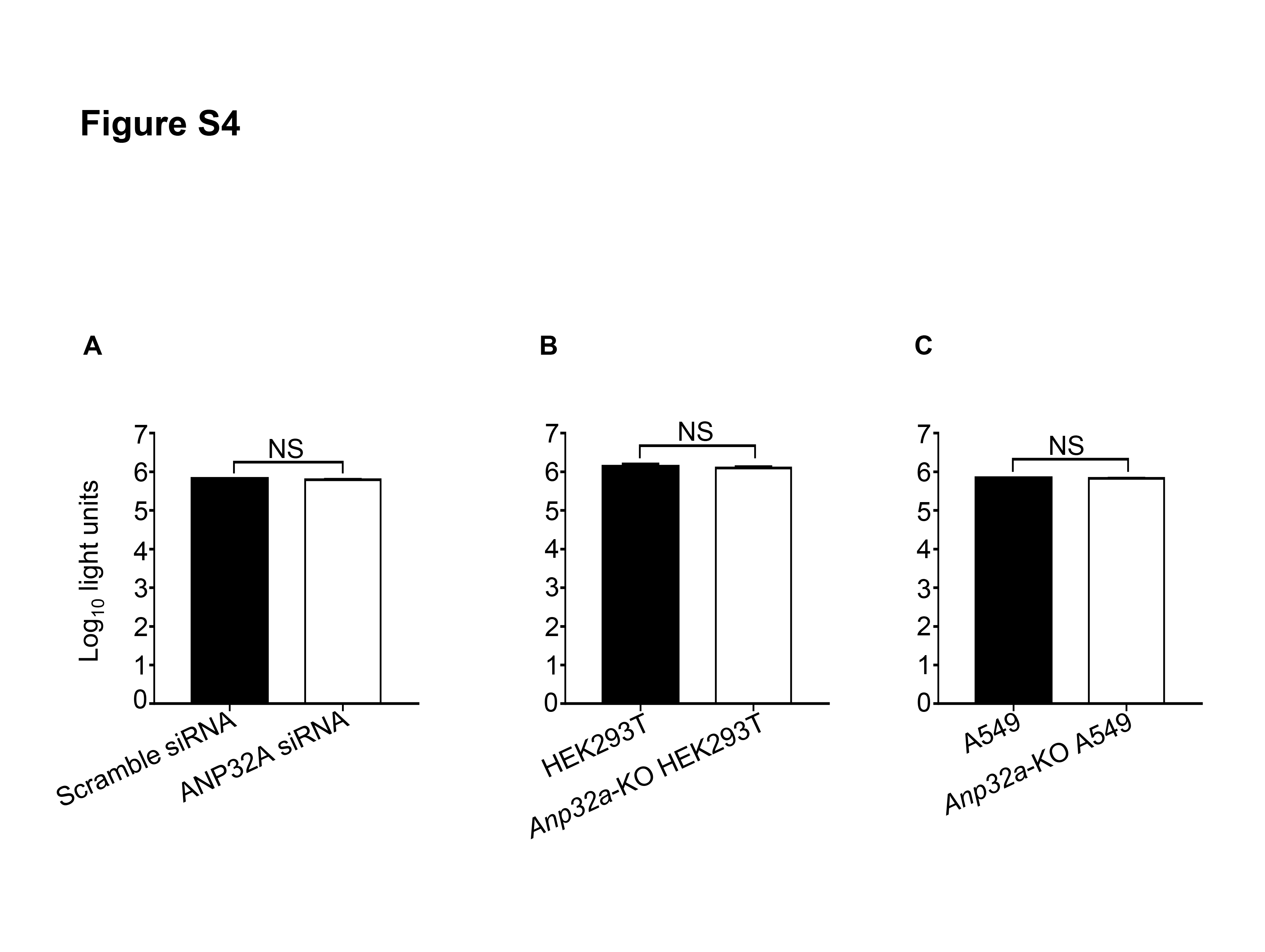

Supplement: FIG S4 [file mBio.01162-19-sf004.tif]

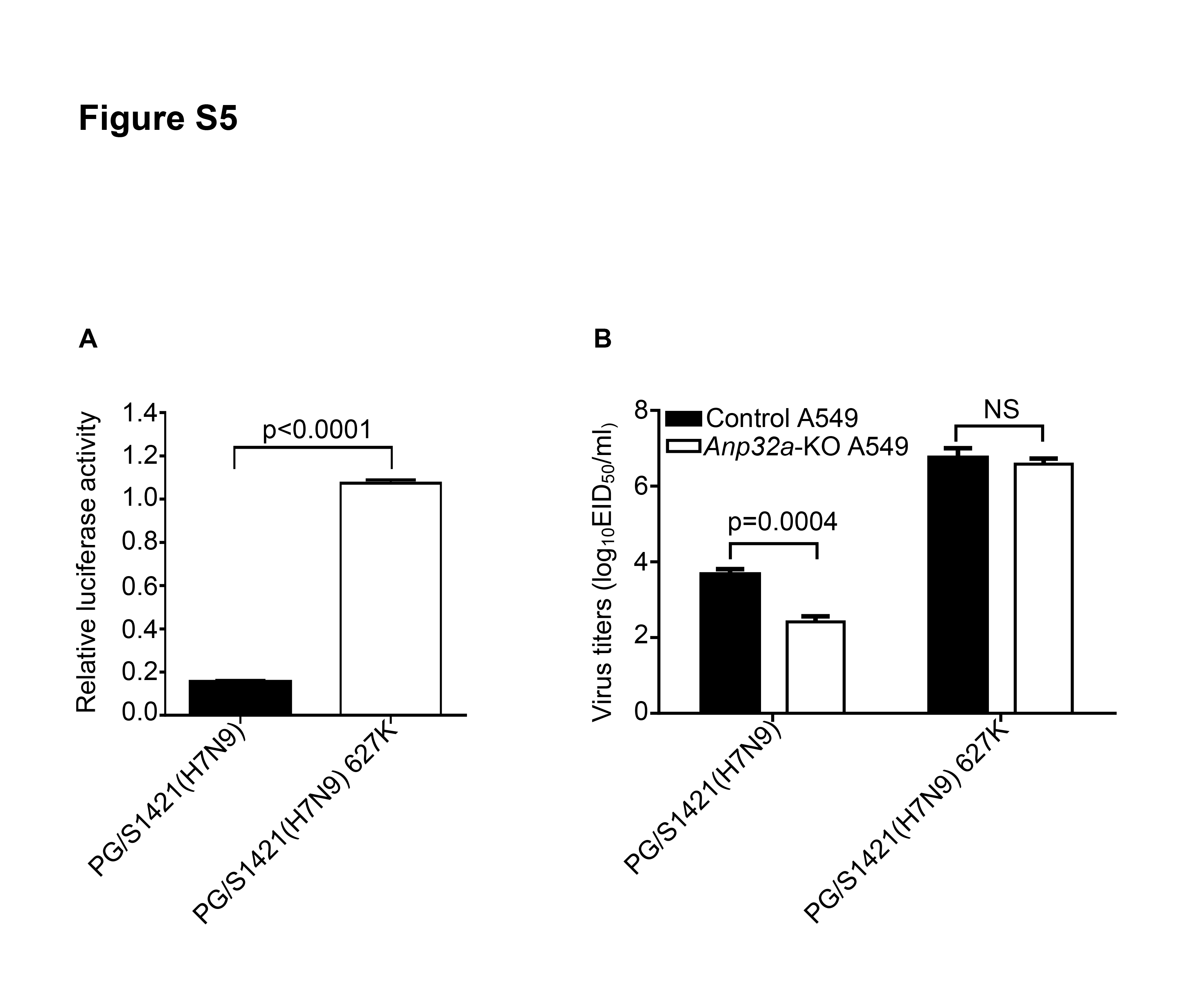

Supplement: FIG S5 [file mBio.01162-19-sf005.tif]

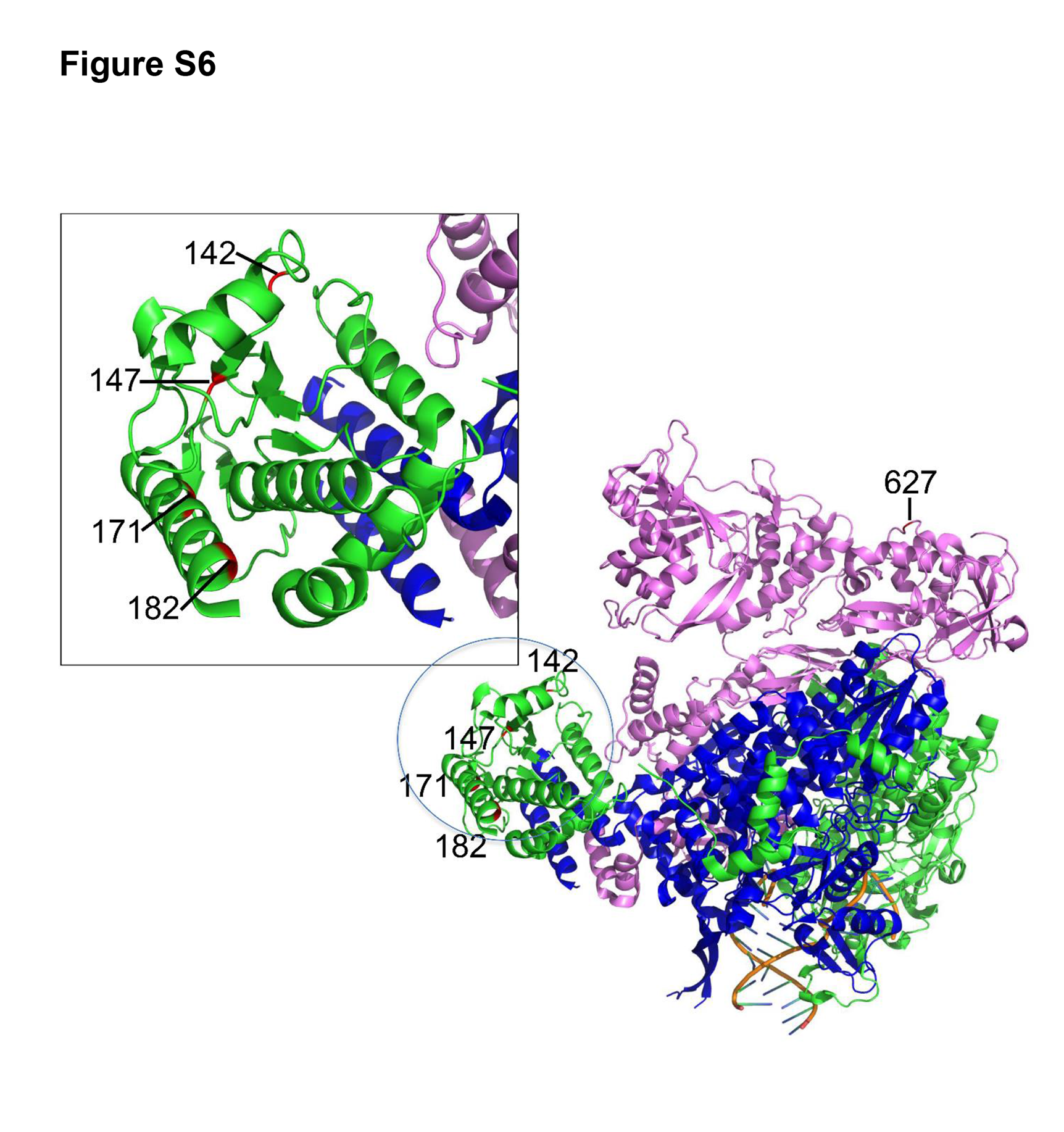

Supplement: FIG S6 [file mBio.01162-19-sf006.tif]
