# Supplementary material for: Low Polymerase Activity Attributed to PA Drives the Acquisition of the PB2 E627K Mutation of H7N9 Avian Influenza Virus in Mammals
Source: mBio. 2019 Jun 18;10(3):e01162-19. doi: 10.1128/mBio.01162-19 (PMC6581862; doi:10.1128/mBio.01162-19)
Supplement: TABLE S2 [file mBio.01162-19-st002.pdf]

Table S2

Virus	Phenotype of the PB2 627 residue during passage in mice[a]
PG/S1421-CK/5PB1+PA(H7N9)	E (3/3)
PG/S1421-CK/5PB2+PB1(H7N9)	K (3/3)
A/chicken/Guangdong/SD008/2017 [CK/SD008(H7N9)]	K (3/3)
CK/SD008-CK/5PA(H7N9)	E (3/3)
CK/SD008-CK/5PA$_{1-191}$(H7N9)	E (3/3)
CK/SD008-PA$_{142R-147V-171V-182L}$(H7N9)	E (3/3)
