# Supplementary material for: Low Polymerase Activity Attributed to PA Drives the Acquisition of the PB2 E627K Mutation of H7N9 Avian Influenza Virus in Mammals
Source: mBio. 2019 Jun 18;10(3):e01162-19. doi: 10.1128/mBio.01162-19 (PMC6581862; doi:10.1128/mBio.01162-19)
Supplement: TABLE S3 [file mBio.01162-19-st003.pdf]

Table S3

Viruses	No. of isolates analyzed	Percentage of PA proteins with the indicated residue (%)									
		142		147		171		182		PG/S1421(H7N9)-like[b]	CK/5(H9N2)-like[c]
		K	R	I	V	I	V	M	L	K-I-I-M	R-V-V-L
Model viruses											
PG/S1421(H7N9)	/	K		I		I		M		/	/
CK/5(H9N2)	/	R		V		V		L		/	/
Avian influenza viruses[a]											
H1	791	98.1	0.88	99.87	0	99.75	0.25	100	0	97.72	0
H2	492	98.17	1.83	100	0	99.8	0.2	100	0	97.97	0
H3	1946	98.68	1.17	99.95	0.05	99.54	0.41	100	0	98.20	0
H4	1761	99.15	0.8	100	0	99.66	0.34	99.89	0	98.69	0
H5	3622	78.16	20.02	99.89	0	99.89	0.06	98.84	1.16	77.91	0
H6	1435	99.09	0.49	100	0	99.86	0.07	100	0	98.95	0
H7	2081	99.33	0.43	100	0	99.81	0.14	99.96	0.04	99.08	0
H8	152	97.37	1.32	100	0	99.34	0.66	100	0	96.71	0
H9	1674	92.83	6.21	99.7	0.12	99.7	0.18	95.1	4.84	90.74	0.12
H10	1005	96.72	2.89	100	0	99	1	100	0	95.72	0
H11	589	99.15	0.51	100	0	99.66	0.34	100	0	98.81	0
H12	192	96.35	3.65	100	0	100	0	100	0	96.35	0
H13	370	99.73	0.07	100	0	99.73	0	100	0	99.46	0
H14	27	100	0	100	0	100	0	100	0	100	0
H15	15	100	0	100	0	100	0	100	0	100	0
H16	222	99.55	0.45	100	0	100	0	100	0	99.55	0
Avian H7N9	648	99.85	0	100	0	100	0	100	0	99.85	0
